# The Resolution of a Cerebrovascular Accident With Phlebotomy in a Hemodialysis Patient With Sickle Cell Disease

**DOI:** 10.7759/cureus.48897

**Published:** 2023-11-16

**Authors:** Azeberoje Osueni, Lutfi Alasadi, Farla Jean-Louis, Shurin Sergiy, Samuel Spitalewitz

**Affiliations:** 1 Nephrology, Brookdale University Hospital Medical Center, Brooklyn, USA; 2 Medicine/Nephrology, Brookdale University Hospital Medical Center, Brooklyn, USA; 3 Internal Medicine, Brookdale University Hospital Medical Center, Brooklyn, USA

**Keywords:** end-stage renal disease (esrd), dialysis, stroke, phlebotomy, sickle cell anemia

## Abstract

Anemia is commonly observed in patients with end-stage renal disease (ESRD) on maintenance hemodialysis (HD) and can be quite severe, particularly when there is an additional comorbidity. With the use of erythropoietin-stimulating agents (ESAs), anemia is effectively treated, but the complete normalization of hemoglobin is not recommended since these agents increase the risk of thrombosis. With improvements in the therapy of sickle cell disease (SCD), patients now survive longer and may more frequently reach end-stage renal disease and require renal replacement therapy. Their anemia can be severe but does respond to ESAs. The goal hemoglobin in these patients is not established and likely should be lower than others on maintenance hemodialysis (HD) since SCD patients already have an increased risk of thrombosis, and the use of ESAs may exacerbate this risk. We present a 57-year-old African-American female with SCD on maintenance HD admitted with an acute cerebrovascular accident (CVA) that occurred in spite of the fact that her hemoglobin was within the accepted range for the general population on maintenance HD. Her neurologic status did not improve with blood pressure control and exchange transfusions, the suggested initial therapy for an acute CVA in a patient with sickle cell disease (SCD). However, with phlebotomy, the patient’s symptoms rapidly improved when her hemoglobin was lowered and subsequently maintained with a lower dose of ESAs. Our experience suggests that the hemoglobin goal in SCD patients on maintenance HD should be lower than in other HD patients. The role of phlebotomy during an acute thrombotic event needs to be explored further.

## Introduction

Anemia is commonly seen in patients on maintenance hemodialysis (HD). This is a result of several factors, the most important of which is the failure of the production of sufficient erythropoietin, which is normally produced in the peritubular cells of the kidney and stimulates red blood cell production. With renal damage, erythropoietin levels fall, resulting in anemia, which can be severe, particularly if there are additional comorbidities [1-6]. Erythropoietin-stimulating agents (ESAs) are now routinely used to treat anemia in patients with end-stage renal disease (ESRD). The goal of anemia treatment with ESAs for patients on dialysis is to mitigate symptoms and reduce the likelihood of needing blood transfusions, which, if performed frequently, can result in iron overload and a myriad of other complications. With the administration of ESAs, most patients on dialysis who have significant anemia (hemoglobin of less than 8 g/dL) experience an improvement in symptoms and report improvement in their quality of life. It is unclear whether the treatment of anemia per se improves patient morbidity, mortality, cardiovascular events, and/or hospitalizations. Since ESAs are known to increase the risk of thrombosis, the recommended hemoglobin level in ESRD patients should be between 10 and 12 g/dL [1,2]. Studies indicate that attempting to achieve higher hemoglobin levels (13 g/dL) with the use of ESAs has been found to be associated with an increased incidence of thrombosis and is not recommended [2].

With more effective therapy for patients with sickle cell disease (SCD), which is frequently associated with a variety of renal disorders and chronic kidney disease (CKD), patients now survive longer and more frequently reach ESRD and require hemodialysis [2-6]. Their anemia is usually more severe than other patients on maintenance hemodialysis. However, it does respond to the administration of ESAs. SCD patients are at higher risk for vascular occlusion and thrombosis, and the administration of ESAs may exacerbate this risk. Although the goal hemoglobin is not well established, it is generally recommended to be less than 10 g/dL, lower than the general population on maintenance hemodialysis.

We present a patient on maintenance HD with SCD hospitalized with an acute cerebrovascular accident (CVA) whose hemoglobin upon presentation was within the accepted goal for the general population on maintenance hemodialysis (10.3 g/dL). Despite blood pressure control and an exchange transfusion, the recommended therapy for an acute CVA in a patient with SCD, there was no improvement in neurologic symptoms. Four serial phlebotomies were then performed, and hemoglobin was maintained at approximately 8 g/dL. With this intervention, the neurologic signs and symptoms rapidly and dramatically improved. The patient’s hemoglobin was maintained at 8-9 g/dL during the hospitalization and after discharge. There was no recurrence of thrombosis. This case report aims to explore the optimal hemoglobin target for SCD patients on maintenance hemodialysis, considering the risks associated with ESAs and the potential benefits of phlebotomy in improving clinical outcomes. By addressing this knowledge gap, we hope to contribute to individualized anemia management strategies and improve patient care for this vulnerable population.

## Case presentation

A 57-year-old African-American female with ESRD on maintenance hemodialysis, SCD, and hypertension was brought to the emergency department with a sudden onset of left-sided weakness. Her vital signs were significant for hypertension (185/98 mmHg) and sinus tachycardia (100 beats per minute {bpm}). On physical examination, she was lethargic and aphasic and did not follow commands. She had a left hemiparesis with a power of 2/5, along with a positive Babinski reflex and a left-sided gaze preference. She was responsive to painful stimuli and only nodded her head when questioned (her Glasgow Coma Scale score was 11). The rest of her physical examination was unremarkable. Prior to the admission, at her outpatient dialysis center, she had received two doses of darbepoetin alfa two weeks apart, and a hemoglobin level of 10.3 g/dL was noted on admission. Her coagulation profile was within normal limits with an international normalized ratio (INR) of 1.04 and a prothrombin time of 11.4 seconds.

**Figure 1:**
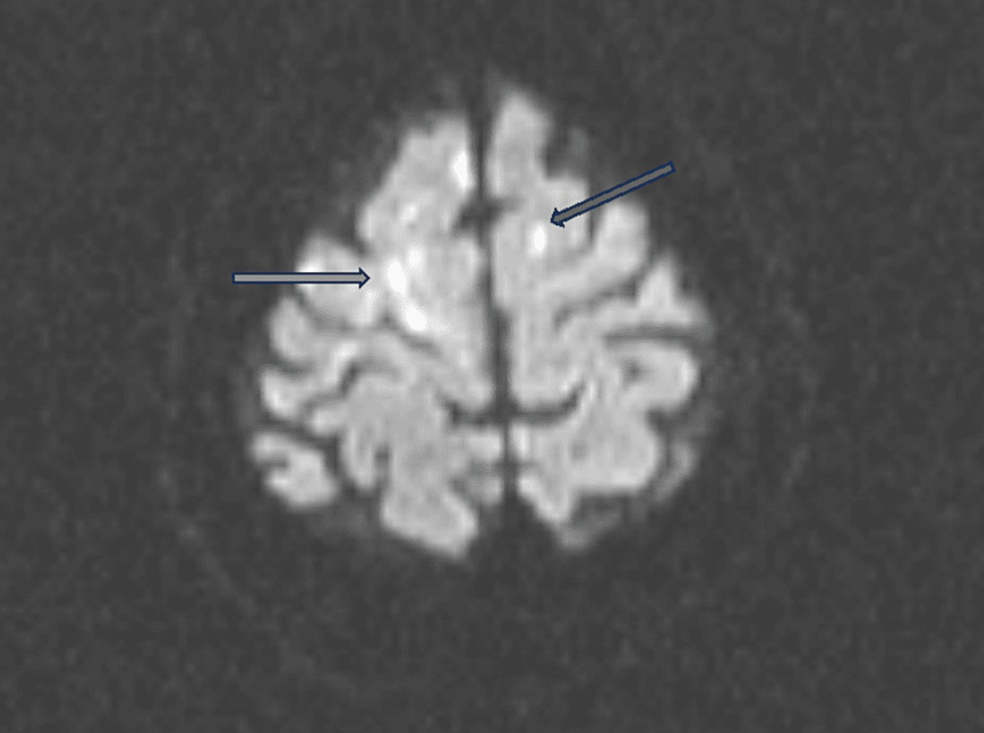
MRI showing multiple small-sized acute infarcts (arrow) MRI: magnetic resonance imaging

A computed tomography (CT) of the head without contrast was done twice within 24-48 hours of admission. Both scans were negative for intracranial hemorrhage and acute infarcts. A CT angiogram of the head and neck was normal. A non-contrast magnetic resonance imaging (MRI) of the brain done within 12 hours after the CT scan, however, showed multiple small-sized acute infarcts within the bilateral watershed territories of the cerebral hemispheres and two acute lacunar infarcts in the cerebellar vermis suggestive of vaso-occlusion (Figure 1).

A small patent foramen ovale (PFO), thought to be clinically insignificant, was identified on a transthoracic echocardiogram. No supraventricular tachyarrhythmia was demonstrated on a Holter monitor.

**Figure 2:**
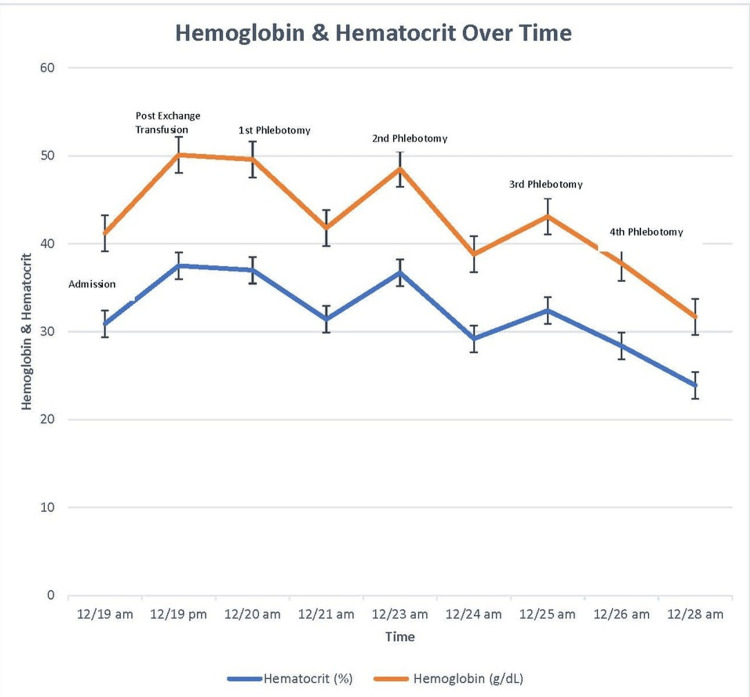
Demonstrating the levels of hemoglobin (Hb) and hematocrit on admission and the changes post exchange transfusion and following phlebotomies The corresponding hemoglobin (Hb) on the Y-axis as calibrated is 3.3, 6.7, 10, 13.3, 16.7, and 20 in ascending order, respectively

An exchange transfusion was done on the same date of admission after which her hemoglobin level increased from 10.3 g/dL to 12.9 g/dL with no neurologic improvement (Figure 2). On the fourth day of admission, to decrease her hemoglobin level, serial phlebotomies were done on four occasions. Five hundred millimeters of blood was removed during each session. Her neurologic status rapidly improved, and the power on the left side increased to 4/5 correlating with the decrement in the hemoglobin level (as shown in Figure 2). The patient continued her hemodialysis sessions and underwent daily physical therapy and at discharge had near-complete resolution of her aphasia and weakness of her left lower extremity.

## Discussion

SCD is the result of a mutation in the beta globulin gene making the hemoglobin less soluble than normal fetal or adult hemoglobin. Due to the increased incidence of vaso-occlusive disease and thrombosis, sickle cell disease has a myriad of clinical manifestations that include painful crises, pulmonary complications, cardiac complications, and liver dysfunction. Frequently devastating are the neurologic complications, the prevalence of which is in the range of 20%-50% [3]. The manifestations vary and may be silent infarctions, acute significant neurologic dysfunction, seizure disorders, and posterior reversible encephalopathy. Clinically significant CVAs are a common and potentially devastating manifestation of SCD [4]. Eleven percent of SCD patients have a clinically evident CVA by 20 years of age and 24% by 45 years of age [5].

Renal involvement is common and affects about 25% of older adults and results in 50% of the deaths from SCD and can lead to ESRD [2]. Since many of the patients expire before requiring renal replacement therapy, the current prevalence of SCD patients on maintenance hemodialysis is estimated to be low at 0.1% but may increase as more effective therapy for SCD becomes available [2].

The pathophysiology of renal injury in SCD begins in the renal medulla and is seen there first because of a combination of the slower medullary renal blood flow inducing hypoxia, acidosis, and hypertonicity. This leads to sickling, which affects the vasculature by thrombosis/vaso-occlusion, obstruction by sickled cells, associated inflammation, vasoconstriction, and the activation of platelets and coagulation factors resulting in hyposthenuria and renal insufficiency. These vascular effects ultimately cause the loss of glomerular and tubular functions [2]. Thus, there are multiple renal manifestations of SCD referred to as “sickle cell nephropathy.” These include, but are not limited to, nephrogenic diabetes insipidus, hyperkalemic renal tubular acidosis, secondary focal glomerulosclerosis with significant proteinuria, renal infarction, and papillary necrosis with renal colic and hematuria, as well as renal medullary carcinoma found almost exclusively in African-Americans with hemoglobin SC disease or sickle trait [3]. As previously stated, ESRD occurs as a result of the above pathophysiologic changes.

The goal hemoglobin in patients with ESRD is between 10 and 12 g/dL [1]. However, there is little data regarding the goal hemoglobin in patients with SCD on dialysis. Because of the higher incidence of thromboses in this patient population, it has been suggested that the level of hemoglobin should be kept lower than generally recommended for others with ESRD (≤10 g/dL) [2]. Our patient presented with a hemoglobin otherwise at “goal” (10.3 g/dL). However, at this hemoglobin level, she suffered a CVA.

Anemia is common in hemodialysis patients, and its presence increases morbidity and mortality [6]. The use of ESAs in dialysis patients is indicated when the hemoglobin level is between 9 and 10 g/dL, and its use may decrease the need for blood transfusion, improve the quality of life and exercise tolerance, and decrease left ventricular hypertrophy [7]. However, the use of ESAs is associated with an increased risk of thrombosis and hypertension and is therefore administered cautiously [8].

ESAs should not be used to maintain a hemoglobin concentration at normal levels in the general ESRD population, since full anemia correction leads to more deaths and myocardial infarctions [9]. On admission, although at the recommended target hemoglobin for the general population with ESRD, our patient presented with a CVA, perhaps because SCD predisposes to such an event through a variety of mechanisms, particularly at higher hemoglobin levels. In addition, the rate of ultrafiltration may further predispose to acute thromboses and neurologic manifestations in patients with SCD on hemodialysis. Because of the limitations of time on hemodialysis and the necessity of removing the volume gained between treatments, the necessary relatively rapid rate of ultrafiltration may also increase the incidence of vaso-occlusive crisis.

As per recommendations for the treatment of acute cerebral thrombosis in sickle cell patients, an exchange transfusion was performed. Unfortunately, there was no improvement in the patient’s central nervous system findings. Thus, as suggested by Bouchaïr et al. [10], we began phlebotomy, and after four sessions, the nadir hemoglobin achieved was 7.3 g/dL. The improvement of her neurologic status including the left-side strength and aphasia correlated with the lowering of the hemoglobin, which was then maintained at approximately 8-9 g/dL as an outpatient. She remained asymptomatic and free of recurrent thrombosis.

As described above, sickle cell nephropathy has a myriad of clinical manifestations ranging from microscopic albuminuria to ESRD. Unfortunately, as compared with other hemodialysis patients, survival is shorter with a mean survival of 54 months [11]. Because many patients do not survive to reach renal replacement therapy, specific data regarding the management of these patients on hemodialysis is scarce [7]. Thus, to our knowledge, there are no different recommendations specifically applicable to SCD patients on hemodialysis regarding the usual parameters such as the adequacy of dialysis, achieving estimated dry weight, optimizing calcium and phosphorus, the control of secondary hyperparathyroidism, vitamin D supplementation, and the use of calcimimetics. The critical role of standardized recommendations will be important in managing these patients. There may be multiple painful crises perhaps related to the rate of ultrafiltration, which should be slowed as much as possible. Because of the slower rate of ultrafiltration, peritoneal dialysis has been suggested as an alternative to hemodialysis. However, there is no data regarding the incidence of painful crises in one modality versus the other. During crises, hemolysis with resultant hyperkalemia needs to be treated expectantly and aggressively. It is not known whether or not access thromboses are more common in these patients.

There appears to be hyporesponsiveness to erythropoietin. This complicates the fact that the utilization of intravenous iron and transfusion should generally be avoided in these patients who have been previously transfused and are frequently iron-overloaded. Further investigations into the role of phlebotomy during acute thrombotic events are warranted to enhance our understanding of tailored anemia management in this patient population.

Limitation

However, this single case report and its objective are limited by the case’s small sample size (single case observation and the lack of a control group). Further case reports or research with a larger patient population and comparative groups is necessary to establish a more robust evidence base for the proposed recommendations in the concluding segment.

## Conclusions

In conclusion, the goal hemoglobin in SCD patients on maintenance hemodialysis needs to be individualized and lower than others with ESRD. The role of phlebotomy during an acute thrombotic episode should be further explored. The treatment of anemia with ESAs is well accepted in patients with ESRD. Their use improves anemia-related symptoms and decreases the need for blood transfusions, which may lead to unwanted iron overload, particularly in patients with SCD. Although the generally recommended hemoglobin level in ESRD is between 10 and 12 g/dL, perhaps, as our experience with this patient suggests, the hemoglobin goal should be significantly less than 10 g/dL in patients with ESRD complicated by SCD. With the judicious use of ESAs, the patient’s hemoglobin level is now being maintained at approximately 8-9 g/dL. There has been no recurrence of CVA, nor are there symptoms of anemia. The treatment of thrombosis with exchange transfusion, if unsuccessful, can be followed by phlebotomy, which, in our patient, clearly improved symptomatology.

## References

[1] Manns BJ, Tonelli M (2012). The new FDA labeling for ESA--implications for patients and providers. Clin J Am Soc Nephrol.

[2] Boyle SM, Jacobs B, Sayani FA, Hoffman B (2016). Management of the dialysis patient with sickle cell disease. Semin Dial.

[3] Ataga KI, Saraf SL, Derebail VK (2022). The nephropathy of sickle cell trait and sickle cell disease. Nat Rev Nephrol.

[4] Vichinsky E (2017). Chronic organ failure in adult sickle cell disease. Hematology Am Soc Hematol Educ Program.

[5] Ohene-Frempong K, Weiner SJ, Sleeper LA (1998). Cerebrovascular accidents in sickle cell disease: rates and risk factors. Blood.

[6] Khan S, Pereira BJ (2000). Hematocrit level associated mortality in hemodialysis patients, by Ma JZ, Ebben J, Xia H, Collins AJ. J Am Soc Nephrol 10:610-619, 1999. Semin Dial.

[7] Drüeke TB, Parfrey PS (2012). Summary of the KDIGO guideline on anemia and comment: reading between the (guide)line(s). Kidney Int.

[8] Coyne DW, Goldsmith D, Macdougall IC (2017). New options for the anemia of chronic kidney disease. Kidney Int Suppl (2011).

[9] (2012). Chapter 3: Use of ESAs and other agents to treat anemia in CKD. Kidney Int Suppl (2011).

[10] Bouchaïr N, Manigne P, Kanfer A (2000). [Prevention of sickle cell crises with multiple phlebotomies] (Article in French). Arch Pediatr.

[11] Alkhunaizi AM, Al-Khatti AA, Al-Mueilo SH, Amir A, Yousif B (2017). End-stage renal disease in patients with sickle cell disease. Saudi J Kidney Dis Transpl.

